# Exposure to antithyroid drugs and ethylenethiourea and risk of thyroid cancer: a systematic review of the epidemiologic evidence

**DOI:** 10.1097/CEJ.0000000000000658

**Published:** 2021-01-22

**Authors:** Carlo La Vecchia, Federica Turati, Eva Negri

**Affiliations:** a Department of Clinical Sciences and Community Health, University of Milan; b Unit of Medical Statistics and Biometry, Fondazione IRCCS Istituto Nazionale Dei Tumori Di Milano; c Department of Biomedical and Clinical Sciences, Università degli Studi di Milano, Milan, Italy

**Keywords:** antithyroid drugs, epidemiology, ethylenethiourea, risk factors, systematic review, thyroid cancer

## Abstract

**Introduction:**

The thyroid peroxidase inhibiting compounds methimazole, methylthiouracil, propylthiouracil, thiouracil (i.e. ‘antithyroid’ drugs) and ethylenethiourea have been associated to thyroid tumours in rodents. According to a systematic review by the International Agency for Research on Cancer (IARC) published in 2000, evidence for the human carcinogenicity was inadequate.

**Methods:**

We performed an up-to-date systematic review of human epidemiological studies on the association between such compounds and thyroid cancer incidence or mortality.

**Results:**

The literature research (1999–March 2020) identified four relevant articles. Considering also reports from the previous IARC review, this systematic review considered seven reports (five distinct studies) on antithyroid drugs and two on ethylenethiourea. As for antithyroid drugs, three reports based on different follow-ups gave results from a cohort of patients treated for hyperthyroidism in 1946–1964. In the earlier report, thyroid cancer incidence was higher in patients primarily treated with antithyroid drugs (3.2/1000) than in those originally treated with thyroidectomy (0.34/1000) or radioactive iodine (0.88/1000), which can be explained by the higher frequency of subsequent thyroidectomy, and hence the higher chance of cancer detection, in that group (30 vs. 0.5 and 1.2%). The two subsequent reports found no deaths from thyroid cancer among patients treated exclusively with antithyroid drugs through 1990 and 2014. A nested case-control study found an odds ratio (OR) of thyroid cancer of 2.79 [95% confidence interval (CI), 0.78–10.02, from a 2-year lag analysis] for ≥3 vs. no propylthiouracil prescriptions. The increased risk can be attributed to advanced diagnosis of an underlying cancer, as suggested by the stronger association observed in a no-lag analysis (OR, 8.03). In a historical cohort of newly diagnosed hyperthyroid patients, the hazard ratio for treatment with radioactive iodine vs. thionamides only was 0.45 (95% CI, 0.21–0.99), possibly due to the closer surveillance of patients receiving thionamides only. Two case-control studies did not find any association with the use of antithyroid drugs. As for ethylenethiourea, no thyroid cancer cases were found in a historical cohort of 1929 workers occupationally exposed in a 15-year period and no association with proxies of mancozeb exposure (a fungicide whose main metabolite is ethylenethiourea) was detected in a cohort of >236 000 farmers.

**Conclusion:**

There is no evidence for a relevant role of either antithyroid drugs or ethylenethiourea on thyroid cancer.

## Abstract 2

Supplemental Digital Content is available in the text.

## Introduction

Thyroid cancer is the ninth cancer in terms of incidence worldwide, with about 430 000 cases in women (incidence rate: 10.2 per 100 000) and 131 000 in men (incidence rate: 3.1 per 10 000) in 2018. In women, it accounts for 5.1% of the total estimated female cancer burden. Mortality rates for the disease are much lower (0.5 in men, and 0.4 in women) (Bray *et al.*, 2018). Approximately two-thirds of all thyroid cancer are papillary carcinomas; other subtypes include follicular carcinomas (10–20%) and medullary carcinomas (5–10%); anaplastic carcinomas, which have the poorest prognosis, are more rare (<5%).

The incidence of thyroid cancer has been largely increasing over the last few years, essentially due to improved diagnostic attention towards largely silent lesions. In contrast, mortality has long been declining (La Vecchia *et al.*, 2015). However, a plateau or some modest rise in mortality has been observed over the last decade in the USA and Europe (La Vecchia and Negri, 2017).

Beside female sex, well-defined risk factors for thyroid cancer are ionizing radiation, benign thyroid disease, iodine imbalance, and familial and genetic factors (La Vecchia *et al.*, 2018). Overweight/obesity, adult height, selected dietary aspects and hormonal and reproductive factors may also have a small role.

In 2001, the International Agency for Research on Cancer (IARC) published the results of Monograph 79, in which evaluated the carcinogenic risk to humans of 19 chemicals that have produced thyroid tumours in rodents, including selected drugs, pesticides, industrial chemicals and a few other compounds (International Agency for Research on Cancer, 2001). These included selected thyroid peroxidase (TPO) inhibiting compounds such as the so-called antithyroid agents methimazole (also known as thiamazole), methylthiouracil, propylthiouracil and thiouracil and the industrial chemical ethylenethiourea. Based on three distinct epidemiologic studies [4 reports (Dobyns *et al.*, 1974; Ron *et al.*, 1987; Hallquist *et al.*, 1994; Ron *et al.*, 1998)] on unspecific antithyroid drugs and 1 study on occupational exposure to ethylenethiourea (Smith, 1976), the IARC working group concluded for all those substances that there was inadequate evidence for the carcinogenicity in humans. In the final evaluation, methimazole and ethylenethiourea were categorized as group 3 (i.e. not classifiable), and methylthiouracil, propylthiouracil and thiouracil as group 2B carcinogens (i.e. possibly carcinogenic). Since the publication of the IARC Monograph, additional evidence on the topic has emerged.

We conducted, therefore, an up-to-date systematic review of the epidemiological evidence in humans on exposure to antithyroid drugs and ethylenethiourea and thyroid cancer risk.

## Methods

Following the guidance on Conducting Systematic Reviews and Meta-Analyses of Observational Studies of Etiology (Dekkers *et al.*, 2019), we conducted a systematic review of human studies investigating the association between exposure to antithyroid drugs or ethylenethiourea and the risk of thyroid cancer.

Eligible studies were human epidemiological studies (case-control studies, cohort studies and cross-sectional studies) investigating the association between methimazole, methylthiouracil, propylthiouracil, thiouracil, unspecified antithyroid drugs or ethylenethiourea and the risk of, or mortality from, thyroid cancer. Studies on exposure to specific fungicides/pesticides whose main metabolite is ethylenethiourea (mancozeb and metiram) and thyroid cancer were also considered. Exclusion criteria were: studies not in humans, reports without original data (e.g. reviews, commentaries and expert opinions), studies without a comparison group (including case reports), studies investigating survival from thyroid cancer and studies investigating unspecified pesticides/fungicides.

We included studies identified in the IARC review (International Agency for Research on Cancer, 2001) and conducted a systematic literature research of additional studies on the topic published from 1999 onwards. The literature research was conducted on 5 March 2020 using *MEDLINE* (through *PubMed*) and *Embase*, without language restriction. The strings included terms for specific compounds (i.e. methimazole, methylthiouracil, propylthiouracil and thiouracil) and antithyroid medications in general, as well as terms for the identification of epidemiological studies on thyroid cancer, without any reference to specific exposures (Supplementary Table 1, Supplemental digital content 1, http://links.lww.com/EJCP/A319). The latter strategy was applied with the aim of identifying publications with data of interest in which specific terms for antithyroid drugs were not reported in the title or the abstract. Additional, simplified, literature researches were conducted in *Google Scholar*, *Web of Science* and the *Cochrane Library*, with similar keywords. We also conducted a supplementary research in *PubMed* to identify original studies investigating exposure to mancozeb/metiram, that is, carbamate fungicides whose main metabolite is ethylenethiourea, in association with thyroid cancer. Two of the authors (E.N. and F.T.) independently screened for inclusion the articles identified through the literature searches, hand-searched the references of the retrieved articles and screened key publications and reviews on the topic.

The same authors extracted independently the characteristics and relevant results of the identified studies using a predefined datasheet. The following data were retrieved: study design, country, type of population, number of subjects considered, number of thyroid cancer cases/deaths, type of exposure and number of exposed subjects, years of study conduction (case-control studies) or period of enrollment and follow-up (cohort studies), quantitative results for the association between the exposure of interest and the risk of thyroid cancer. Whenever available, results adjusted for potential confounders were considered.

## Results

**Fig. 1:**
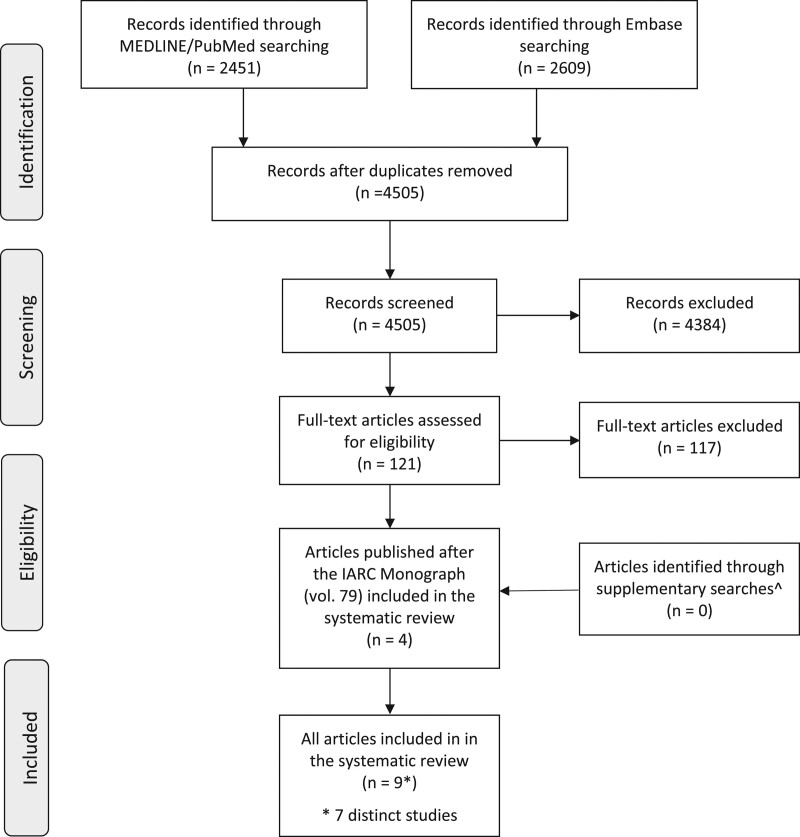
Flowchart of the study selection procedure (date span of search: 1999 to 5 March 2020). Additional, simplified searches in *Google Scholar*, *Web of Science* and the *Cochrane Library* and a supplementary search in PubMed specific for mancozeb and metiram.

The flowchart of the study selection procedure is reported in Fig. 1. Among the 4505 unique records identified in *MEDLINE*/*PubMed* and *Embase* (date span of the search: 1999 to 5 March 2020), four articles satisfied the inclusion criteria and were finally included in the systematic review. No further eligible studies were identified through additional searches in *Google Scholar*, *Web of Science* and the *Cochrane Library* databases and the supplementary search including specific terms for mancozeb and metiram as well as through the screening of the bibliographies of the included reports.

### Description of the individual studies

Of the four identified articles (Nordby *et al.*, 2005; Friedman *et al.*, 2009; Tulchinsky and Brill, 2019; Gronich *et al.*, 2020), three investigated antithyroid drugs (Friedman *et al.*, 2009; Tulchinsky and Brill, 2019; Gronich *et al.*, 2020) (two unspecified antithyroid drugs and one propylthiouracil) and one study focused on exposure to the fungicide mancozeb (Nordby *et al.*, 2005). Considering also reports already included in the review by IARC (International Agency for Research on Cancer, 2001), the present systematic review included seven reports (five distinct studies) on exposure to antithyroid drugs and thyroid cancer and two reports on exposure to ethylenethiourea and thyroid cancer.

Herein, we describe in the chronological order and by study design the main characteristics and results of all these studies, separately for antithyroid drugs and ethylenethiourea. Details of studies are provided in Table 1 for cohort studies and Table 2 for case-control studies on exposure to antithyroid drugs and thyroid cancer, and in Table 3 for cohort studies on exposure to ethylenethiourea and thyroid cancer.

**Table 1:** Cohort studies of thyroid cancer and exposure to antithyroid drugs

Reference	Population, follow-up	Exposure	No. of thyroid cancers cases/deaths	Results
Cooperative thyrotoxicosis Therapy Follow-up study, US and UK	Cohort study of patients treated for hyperthyroidism^a^ between 1946 and1968 in 25 US and 1 UK medical centers			
Dobyns *et al.* (1974) ^b^	Overall cohort: 34 684Follow-up: through 1968 (median time 8.2 years)	1238 patients primarily treated at study inclusion with unspecified antithyroid drugs for ≥1 year	Among patients treated with antithyroid drugs for at least 1 year:0 thyroid cancers within 1 year of treatment;4 thyroid cancers 1 year or more after study entry	Crude incidence rate/1000 of thyroid cancer 1 year or more after the start of the study: 3.2 in patients originally treated with antithyroid drugs for at least 1 year 0.34 in patients originally treated with radioactive iodine (*n* = 21 714);
	
		
				0.88 in patients in patients originally treated with thyroidectomy (*n* = 11 732);
Ron *et al.* (1998) ^b^	Overall cohort: 35 593Follow-up: through 1990 (median time 21 years)	1374 patients treated with antithyroid drugs only	Among patients treated with antithyroid drugs only:	Expected number of deaths from thyroid carcinoma not reported
		(1177 considered for the SMR analyses)	0 deaths from thyroid cancer 1 year or more after the start of the study	
Tulchinsky and Brill 2019	Overall cohort: 31 332^c^ Follow-up: through 2014^c^	No. of patients treated with antithyroid drugs only: NA	Among patients treated with antithyroid drugs only:	Expected number of deaths from thyroid carcinoma not reported
			0 deaths from thyroid cancer (1 year or more after the start of the study^d^)	
Friedman *et al.* (2009), US	Nested case-control study (1994–2006) based on a cohort of 6 465 309 subscribers of a medical care program	4252 individuals with propylthiouracil prescriptions	Among individuals with ≥3 propylthiouracil prescriptions:3 thyroid cancers	OR for ≥3 prescription vs. no use at least 2 years before cancer diagnosis 2.79 (95% CI, 0.78–10.02)
	Follow-up: 12 years			
Gronich *et al.* (2020), Israel	Historical cohort of 16 637 patients with a new diagnosis of hyperthyroidism in 2002–2015 identified from a health services provider	13 808 patients treated with thionamides (propylthiouracil or thiamazole) only	Among patients treated with thionamides only: NAAmong the overall cohort:74 thyroid cancers	Crude incidence rate/100 000 (95% CI) and hazard ratio (95% CI) of thyroid cancer:Incidence rate – thionamide only: 6.59 (5.18–8.37)Incidence rate – any radiodine^e^: 3.26 (1.56–6.84)Hazard ratio^f^ (radiodine vs. thionamide): 0.45 (0.21–0.99)
	Follow-up: through mid-2016 (mean time: 7.3 years)			
		
		

CI, confidence interval; NA, not available; OR, odds ratio; SMR, standardized mortality ratio.

a About 40% of patients had received previous therapy for hyperthyroidism before study inclusion.

b Included in the IARC monograph, volume 79.

c Information retrieved from Khitara *et al*.

d Deduced because the methods were declared similar to those of Ron *et al*.

e Radioactive iodine with or without other treatments for hyperthyroidism.

f Adjusted for the propensity score of receiving radioactive iodine, which took into account age, sex, smoking history, BMI, district, socioeconomic status, diabetes mellitus, hypertension, pharmacy administration of aspirin and of statins, and adherence to mammography and to fecal occult blood test.

**Table 2:** Case-control studies of thyroid cancer and exposure to antithyroid drugs

Reference	Study design	Cases/controls	Exposed	Results
Ron *et al.* 1987^a^, US	Population-based case-control study in 1978–1980	159 cases of thyroid cancer and 285 controls	NA	No association with antithyroid drugs (OR not reported)
Hallquist *et al.* 1994^a^, Sweden	Population-based case-control study in 1980–1989	180 cases of thyroid cancer and 360 controls	Among antithyroid-drugs users: 2 cases and 2 controls	OR for antithyroid drugs use: 2.0 (95% CI, 0.2–21)

CI, confidence interval; NA, not available; OR, odds ratio.

^a^Included in the IARC monograph, volume 79.

**Table 3:** Cohort studies on thyroid cancer and exposure to ethylenethiourea

Reference	Population, follow-up	Exposure	No. of thyroid cancers cases/deaths	Results
Smith 1976^a^, England	Retrospective cohort of 1929 ethylenethiourea-exposed workers identified through employment records Follow-up: 1957–71	1929 ethylenethiourea-exposed workers	0 thyroid cancers	0 thyroid cancers in the cohort
Nordby *et al.* 2005, Norway	Cohort of 141 389 farm holders (born in 1925–1971) and 95 257 spouses to farm holders (and their 286 475 children) identified from a population register and agricultural censuses in 1969–1989 Follow-up: through 2000	Proxies of mancozeb exposures: potato farming, pesticide use and fungal forecasts in 1973–1990No. in potato farming: 323 183 No. in pesticide use: 207 907 No. in the highest category of number of fungal forecasts in 1973–1990: 141,903	No. of thyroid cancers: Potato farming: 183 Pesticide use: 118 Highest category of fungal forecasts in 1973–1990: 92	Crude incidence rate/100 000 (95% CI) and rate ratio^b^ (95% CI) of thyroid cancer Potato farming: Incidence rate – No: 4.27 (3.61–5.06) Incidence rate – Yes: 3.25 (2.82–3.76) Rate ratio (yes vs no): 0.87 (0.69–1.19) Pesticide use: Incidence rate – No: 3.78 (3.29–4.34) Incidence rate – Yes: 3.38 (2.82–2.05) Rate ratio not reported Fungal forecasts in 1973–1990: Incidence rate – 0 seasons: 3.82 (3.05–4.79) Incidence rate – 1–2 seasons: 3.26 (2.63–4.05) Incidence rate – 3–5 seasons: 3.55 (2.81–4.50) Incidence rate – 6–13 seasons: 3.90 (3.18–4.78) Rate ratio (6–13 seasons vs 0): 1.27 (0.83–1.93)

CI, confidence interval.

^a^Included in the IARC monograph, volume 79.

b Adjusted for age, gender, birth year, region and adjusted for other proxies of mancozeb exposure.

### Antithyroid drugs

#### Cohort studies

The Cooperative Thyrotoxicosis Therapy Follow-up Study included over 35 000 patients treated for hyperthyroidism in 25 medical centers in USA and 1 in England between 1946 and 1964. About 40% of the study participants had received some form of therapy for hyperthyroidism before study inclusion, mostly antithyroid drugs or radioactive iodine or a combination of the two. Three publications provided results on treatment with antithyroid drugs and thyroid cancer.

In the 1974 publication by Dobyns *et al*. (Dobyns *et al.*, 1974), 34 684 patients were classified according to the first primary treatment given in the study and were followed-up until 1968, with a median follow-up of 8.2 years. Of the 1238 patients with hyperthyroidism primarily treated with unspecified antithyroid drugs for at least 1 year, 374 were ultimately treated with radioactive iodine, 356 by thyroidectomy and the remaining 508 did not receive any other form of therapy for hyperthyroidism during follow-up. No malignant thyroid neoplasm was found within 1 year of antithyroid drugs therapy and four malignant thyroid neoplasms were detected after an interval of 1 or more years from study entry, corresponding to a rate of 3.2/1000 patients. The corresponding rates/1000 for patients originally treated with thyroidectomy and radioactive iodine were, respectively, 0.34 (4 malignant tumors in 11 732 patients) and 0.88 (19 malignant tumors in 21 714 patients). Authors recognized relevant limitations in comparing the occurrence of malignant tumors across the three cohorts; these included the differences in age, duration of the observation and frequency of subsequent thyroidectomy (higher in patients treated with antithyroid drugs) across treatment groups, the fact that nodules and potential nodules were removed in patients initially treated with thyroidectomy, the probable presence of clinically unrecognized nodules in some patients, and the differences in the frequency of thyroid nodules not palpable at study entry but present and not removed at study closure.

Ron *et al.* (1998) updated in 1998 the report of Dobyns *et al.* (1974) by conducting a second mortality follow-up through 1990 of the original cohort. After reassembling the cohort, the study population comprised 35 593 individuals (79% women). During a median follow-up of 21 years, about 19% of patients were lost and 50.5% of the study participants died. A total of 1374 patients were treated with antithyroid drugs only, 10 439 with radioactive iodine and drugs, 10 381 with thyroidectomy and drugs, 2661 with a combination of the three types of treatment and the remainder by other means. The drugs used during the study period were chiefly thiourea derivatives and iodine compounds. The standardized mortality ratios (SMR) were calculated on 33 748 patients from the USA with available information on sex, date of birth and date of study entry; of these, 1177 were treated exclusively with antithyroid drugs. One year or more after the start of the study, no deaths from thyroid cancer were recorded in the antithyroid drugs treatment group. The expected number of deaths from thyroid carcinoma was not reported (probably, it was less than 1). The SMR for thyroid carcinoma death were 3.94 [95% confidence interval (CI), 2.52–5.86; 24 observed deaths] in patients treated with radioactive iodine with or without drugs (*n* = 20 949), 4.91 (95% CI, 2.45–8.79; 11 observed deaths) in those treated with radioactive iodine only (*n* = 8054) and 1.07 (95% CI not available, four observed deaths) in patients with surgery with or without drugs (*n* = 10 876). In discussing some excess risk for selected nonthyroid cancers, the authors acknowledged as limitations the small sample size of the group treated with drugs only and the lack of information on the type, quantity and dates of drug use; they also recognized the higher prevalence of prior cancer in that treatment group, with the consequent possible selection of patients with health problems for drug therapy.

Extended follow-up data through 2014 [information retrieved from Khitara *et al*. (2019)] were provided by Tulchinsky and Brill (2019) in a commentary article. The two authors criticized an investigation by Khitara *et al*. (2019) (which they co-authored), based as well on the Cooperative Thyrotoxicosis Therapy Follow-up Study. The article by Khitara *et al*. (2019) showed an excess risk of death from solid cancers and specifically from breast cancer for greater organ-absorbed doses of radioactive iodine. According to Tulchinsky and Brill (2019), the investigation by Khitara *et al*. (2019), which focused only on patients receiving radioactive iodine therapy (*n* = 18 805), did not compare the risks of such therapy with the risks associated to other hyperthyroidism treatments, providing therefore inconsistent evidence on the possible carcinogenicity of radioactive iodine treatment. Using the same population as in Khitara *et al*. but including also patients with hyperthyroidism therapies other than radioactive iodine, for a total of 31 332 patients [information retrieved from Khitara *et al*. (2019)], the authors provided the SMR of various cancers according to different patterns of hyperthyroidism treatments. One year of more after study entry [information deduced because the methods were declared similar to those of Ron *et al.* (1998)], no deaths from thyroid cancer were observed among patients treated exclusively with antithyroid drugs (number of patients and observed/expected deaths not provided), and no SMR was calculated. Elevated SMRs of thyroid cancer were observed for treatment with radioactive iodine only (3.09; 95% CI, 1.61–5.3; based on 11 observed deaths) and radioactive iodine with or without other treatments (3.35; 95% CI, 2.3–4.7; based on 31 observed deaths).

Friedman *et al.* (2009) used a nested case-control approach to investigate the possible human carcinogenicity of nine pharmaceuticals, including propylthiouracil, in a large medical care program with automated pharmacy records [Kaiser Permanente Medical Care Program, (KPMCP)] and a cancer registry, in North California. The cohort included 6 465 309 KPMCP subscribers, who were followed for 12 years. A total of 4252 subjects were prescribed propylthiouracil. During the follow-up period, 131 743 subjects developed at least one incident cancer in the overall cohort and 2072 developed a thyroid cancer. For each case patient, 10 controls without the cancer of interest at the date of the case’s diagnosis and matched for sex, age and year of starting drug coverage were randomly selected from the cohort. The odds ratio (OR) of thyroid cancer for three or more prescriptions of propylthiouracil vs. no use was 2.79 (95% CI, 0.78–10.02). The estimate was obtained from a 2-year lag analysis, which considered only exposures occurred at least 2 years before the date of cancer diagnosis and which was based on three thyroid cancers among propylthiouracil-exposed subjects. In a no-lag analysis (including also drug prescription in the 2 years before cancer detection), the OR increased to 8.03 (95% CI, 3.50–18.39, based on 10 thyroid cancer cases), which suggested, as also stated by the authors, a role of diagnostic bias in the observed nonsignificant association between propylthiouracil use and thyroid cancer.

Gronich *et al*. conducted a historical cohort study of 16 637 cancer-free patients with a new diagnosis of hyperthyroidism in 2002–2015 who were treated with any thionamide drug (propylthiouracil or thiamazole) or with radioactive iodine identified from a health services provider in Israel (Gronich *et al.*, 2020). Patients were followed through the mid of 2016, for a mean follow-up time of 7.3 years. A total of 13 808 patients were treated with thionamides only and 2829 received radioactive iodine. Among the latter group, 1808 were treated with thionamides before radioactive iodine; such patients contributed to follow-up time of both thionamides and radioactive iodine treatment groups according to the date of administration of radioactive iodine. A total of 74 thyroid cancers were registered in the overall cohort. The crude incidence rates/100 000 were 6.59 (95% CI, 5.18–8.37) for patients treated with thionamides only and 3.26 (95% CI, 1.56–6.84) for radioactive iodine-treated ones. The hazard ratio for treatment with radioactive iodine vs. thionamides only was 0.45 (95% CI, 0.21–0.99), adjusted by the propensity score to receive radioactive iodine (which took into account demographic and clinical factors, including age, sex, smoking, BMI, socioeconomic status, diabetes, hypertension, pharmacy administration of aspirin and of statins, and adherence to recommended cancer screening procedures), indicating no excess risk of thyroid cancer for treatment with radioactive iodine compared to treatment with thionamides only. There was a moderate excess in overall mortality for patients treated with thionamides only, as compared to those treated with radioactive iodine, with a propensity score-adjusted hazard ratio of 1.21 (95% CI, 1.05–1.39). This has however no direct relevance to the thyroid cancer issue.

#### Case-control studies

Ron *et al*. (9) conducted a population-based case-control study including 159 cases of thyroid cancer identified from a cancer registry and 285 population controls in Connecticut, USA, between 1978 and 1980. The use of antithyroid drugs was not associated with an increased risk of thyroid cancer (relative risk not shown).

Hallquist *et al.* (1994) carried out a case-control study on thyroid cancer in Sweden. Between 1980 and 1989, 180 cases of thyroid cancer and 360 population controls matched for age and sex were evaluated. The OR for the use of antithyroid drugs was 2.0 (95% CI, 0.2–21), based on two cases and two controls exposed only.

### Ethylenethiourea

#### Cohort studies

Smith (1976) conducted two investigations in England with the aim of assessing the human teratogenicity and the human thyroid carcinogenicity of ethylenethiourea. With regard to thyroid cancer, the author investigated a historical cohort study of 1929 workers at several large rubber-manufacturing plants where ethylenethiourea was used and at a plant producing ethylenethiourea in England. Workers were identified from employment records. According to the Birmingham Cancer Registry for the period 1957–1971, none of the workers developed thyroid cancer. On the basis of the expected occurrence of thyroid cancer in the general population, the author concluded that it was very encouraging that no cancers occurred in the 1929 employees. The author also reported results from a small survey based on another plant which had used ethylenethiourea since 1952. Although 49 thyroid cancers were registered in 1957–1971 in the town where the plant was located, no thyroid cancer was reported in workers at the plant.

A study of farmers’ families was conducted in Norway to investigate the association between indicators of mancozeb exposure and the risk of thyroid cancer and neural tube defects (Nordby *et al.*, 2005). A cohort of 141 389 farmers born in 1925–1971 and 95 257 spouses to farmers (and their 286 475 children for the analysis on neural tube defects) was conducted through record linkage between the Central Population Register and agricultural censuses conducted in the periods 1969–1989. Data on farm production available from agricultural censuses and meteorologically based fungal forecasts provided the proxies of mancozeb exposure, that is, potato farming, pesticide use (pesticide purchase in 1969 census or spraying equipment in 1979 census) and the number of seasons in 1973–1990 with more than one fungal forecast (at each farm was allocated the number of fungal forecasts from the nearest station). The subjects were followed for thyroid cancer in the Cancer Register through 2000. Overall, a total of 319 cases of thyroid cancer over 8.8 million person-years occurred. There was no association between any proxy of mancozeb exposure and the risk of thyroid cancer. The crude incidence rates for 100 000 person-years were 3.25 (95% CI, 2.82–3.76) for potato farming (183 cases over 323 183 person at risk) and 4.27 (95% CI, 3.61–5.06) for no potato farming (136 cases over 199 938 persons at risk); after allowance for gender, age, birth year, fungal forecasts and region, the rate ratio for potato farming was 0.87 (95% CI, 0.69–1.19). Crude incidence rates for 100 000 person-years were 3.38 (95% CI, 2.82–2.05) for pesticide use (118 cases over 207 907 persons at risk) and 3.78 (95% CI, 3.29–4.34) for no pesticide use (201 cases over 315 214 persons at risk) (no rate ratio was provided). Incidence rates for increasing categories of fungal forecasts were 3.82 (95% CI, 3.05–4.79), 3.26 (95% CI, 2.63–4.05), 3.55 (95% CI, 2.81–4.50) and 3.90 (95% CI, 3.18–4.78); the adjusted rate ratio for the highest category of fungal forecasts vs. no season with >1 fungal forecast was 1.27 (95% CI, 0.83–1.93). Results for potato farming and fungal forecasts were similar when considering papillary thyroid cancer only.

## Discussion

The present review systematically evaluated the possible association between human exposure to selected compounds inhibiting thyroid hormone synthesis via inhibition of the TPO and thyroid cancer incidence and mortality. Based on seven reports (from five distinct studies) on exposure to antithyroid drugs and thyroid cancer and two reports on exposure to ethylenethiourea and thyroid cancer, the review showed no evidence for a relevant role of these compounds on thyroid cancer.

Methimazole, methylthiouracil, propylthiouracil and thiouracil are TPO inhibitors. Ethylenethiourea has TPO-inhibiting properties (Doerge and Takazawa, 1990; Freyberger and Ahr, 2006). The pesticidal active ingredients mancozeb and metiram (dithiocarbamates) are intrinsically metabolized to ethylenethiourea and further small molecules in mammals.

With regard to antithyroid drugs, three reports (Dobyns *et al.*, 1974; Ron *et al.*, 1998; Tulchinsky and Brill, 2019), based on different follow-up periods, gave results for the Cooperative Thyrotoxicosis Therapy Follow-up Study. In the first report (1974) (Dobyns *et al.*, 1974), an apparent direct association between treatment with antithyroid drugs and thyroid cancer was observed, based on the comparison of (crude) incidence rates of thyroid neoplasm across groups of patients classified according to the first primary treatment given in the study. Results were based on a small number of thyroid cancer cases (i.e. 4 in patients primarily treated with antithyroid drugs for ≥ 1 year, 4 in those originally treated with thyroidectomy and 19 in patients originally treated with radioactive iodine) and no adjustment was performed to account for different patients’ characteristics and observation periods across the three treatment groups. Further, about 40% of patients had received previous treatment for hyperthyroidism at study inclusion, and several patients received various therapies during follow-up (e.g. 60% of those in the antithyroid drugs group). The analysis based on the primary treatment given at study inclusion does not allow us to disentangle the effects of the different therapies for hyperthyroidism. As also recognized by the authors, a likely explanation for the excess of thyroid cancer among patients primarily treated with antithyroid drugs is the higher rate of subsequent thyroidectomy in that group (30%) compared to other treatment groups (0.5% in patients initially treated with radioactive iodine and 1.2% in those with primary thyroidectomy), which increased cancer detection. Subsequent reports with extended follow-up focused on patients treated exclusively with antithyroid drugs (with the potential to better disentangle the effect of antithyroid drugs from that of other treatments for hyperthyroidism) and reported no death from thyroid cancer in that group (Ron *et al.*, 1998; Tulchinsky and Brill, 2019). Given the small number of incident cases in the antithyroid drugs group (*n* = 4) and the greater diagnostic detection, which has a major impact on thyroid cancer incidence, the interpretation of the results of the earlier report remains undefined. Considering the null results on mortality of the two more recent reports too, the evidence from the Cooperative Thyrotoxicosis Therapy Follow-up Study (Dobyns *et al.*, 1974; Ron *et al.*, 1998; Tulchinsky and Brill, 2019) is not supportive of a direct association between use of antithyroid drugs and thyroid cancer.

In a cohort study of subscribers of a medical care program (Friedman *et al.*, 2009), there was a positive but non-significant association between ≥3 prescriptions of propylthiouracil and thyroid cancer in a 2-year lag analysis. The small number of events (three thyroid cancers in exposed subjects) and hence the very large CI of the OR (i.e. from 0.78 to 10.02) limit the interpretation of the results. The apparent non-significant association, besides random variation, can be explained by confounding by benign thyroid conditions or earlier diagnosis of underlying thyroid cancer, as acknowledged by the authors. Along this line, a much stronger association was found when considering also propylthiouracil prescriptions in the 2 years before cancer diagnosis.

In a historical cohort of patients with a new diagnosis of hyperthyroidism (Gronich *et al.*, 2020), there was a reduced risk of thyroid cancer, of borderline significance, for treatment with radioactive iodine compared to treatment with thionamides only, based on 74 incident cases. The study was designed to evaluate the carcinogenicity of radioactive iodine treatment, and its relevance to evaluate thionamides is questionable because also in the group using radioactive iodine treatment the majority of patients had used thionamides. In addition, the decreased thyroid cancer risk observed for treatment with radioactive iodine vs. thionamides may be explained by the closer surveillance of patients receiving thionamides (because the hyperthyroid state is not cured as in patients treated with radioiodine), with the consequent earlier detection of indolent tumors (overdiagnosis) in that group (Lortet-Tieulent *et al.*, 2019).

In addition, the two available case-control studies did not find any significant association between use of antithyroid drugs and thyroid cancer (Ron *et al.*, 1987; Hallquist *et al.*, 1994). Thus, according to available epidemiological data, there is no consistent evidence for a relevant role of antithyroid drugs in thyroid cancer.

Two studies gave results on exposure to ethylenethiourea and thyroid cancer (Smith, 1976; Nordby *et al.*, 2005). The first one, using registry data, found no thyroid cancer cases in a cohort of workers occupationally exposed to ethylenethiourea during a 15-year period (Smith, 1976). The second one was a cohort study of Norwegian farmers born in 1925–1971 and their families identified by national registries and agricultural censuses, and found no association between proxies of exposure to mancozeb and the risk of thyroid cancer (Nordby *et al.*, 2005). Mancozeb, which is metabolized to ethylenethiourea, was the most frequently used fungicide in Norway at the time of study conduction, where it has been used mainly in potato cultivation. Major limitations of those studies include the lack of details on study methods and of the expected number of thyroid cancers in the earlier one (Smith, 1976) and the use of proxies for mancozeb exposure with the consequent potential misclassification in the second one (Nordby *et al.*, 2005). Still, the available evidence does not support a relevant role of exposure to ethylenethiourea on thyroid cancer risk.

In conclusion, the present systematic up-to-date review indicates that there is no consistent evidence for a relevant role of antithyroid drugs on thyroid cancer; evidence on ethylenethiourea, albeit limited, does not support a role of this compound on thyroid cancer risk.

## Acknowledgements

This work was supported by an unconditional consulting agreement with BASF SE to the Department of Clinical Sciences and Community Health of the University of Milan.

## Conflicts of interest

There are no conflicts of interest.

## Supplementary Material
