# Supplementary material for: Exposure to antithyroid drugs and ethylenethiourea and risk of thyroid cancer: a systematic review of the epidemiologic evidence
Source: Eur J Cancer Prev. 2021 Jan 22;31(1):64–72. doi: 10.1097/CEJ.0000000000000658 (PMC10883355; doi:10.1097/CEJ.0000000000000658)
Supplement: Supplementary file 1 [file ejcp-31-64-s001.pdf]

Supplementary Table 1. Reporting of the search process for scientific literature in bibliographic databases*.

	Details of the searches	
	**Pubmed**	**EMBASE**
**Date of search**	5 March 2020	5 March 2020
**Date span of the search**	1999 to 5 March 2020	1999 to 5 March 2020
**Search strategies**	1. Methimazole OR Methylthiouracil OR Propylthiouracil OR Thiouracil OR Ethylenethiourea OR "Antithyroid Agents"[Mesh] OR ((Antithyroid OR anti-thyroid OR "anti thyroid" OR anti-thyroidal OR antithyroidal OR "anti thyroidal") AND (drug OR drugs OR agent OR agents)) AND cancer<br>2. ("Thyroid Neoplasms/epidemiology"[Mesh] OR "Thyroid Neoplasms/etiology"[Mesh]) AND (case-control OR cohort) AND risk<br>3. #1 OR #2<br>4. Limit #3 to publication date from 1/1/1999 onwards	1. ('malignant neoplasm'/exp AND ('antithyroid agent'/de OR methimazole OR methylthiouracil OR propylthiouracil OR thiouracil OR ethylenethiourea) OR (('risk'/exp OR risk) AND 'thyroid cancer'/exp AND ('case control study'/de OR 'cohort analysis'/exp OR 'cohort analysis')))<br>2. Limit #1 to publication date from 1/1/1999 onwards
**Total number of summary records retrieved**	2451	2609
**Total number of summary records retrieved after removing duplicates:** 4505		

*Additional, simplified, literature searches with similar keywords were conducted in Google Scholar, Web of Science and the Cochrane Library. A supplementary search in PubMed with keywords for mancozeb and metiram and thyroid cancer was also conducted.
